# Allegheny County Medical Society

**Published:** 1892-07

**Authors:** 


### ALLEGHENY COUNTY MEDICAL SOCIETY.

PITTSBURG, PA., May 10, 1892.

DR. SAMUEL AYRES read the following paper:

### CLINICAL NOTES ON A CASE OF HYSTERIA IN A BOY EIGHT YEARS OF AGE.

MR. PRESIDENT AND GENTLEMEN—The rather infrequent occurrence, or perhaps recognition, of hysteria in young children, induces me to report the following interesting case :

William T., *æt.* eight years, had good family history and previously good health. In the early part of December, 1891, while attending school he became involved in a quarrel with four boys who pitched upon and struck him about the face and head, there being left, however, no visible bruises or marks of injury. Immediately after returning home that evening he complained of severe headache, not specialiy localized. This continued without abatement for about two weeks, when the family physician, Dr. Wallace of Ingram, was consulted. After treating the patient a few weeks there was still no improvement in the headaches, but otherwise the boy's general condition was not much impaired. He had not been allowed to attend school since the accident, but played about the house, had a fair appetite, bowels were regular, and there was no abnormal variation in temperature or pulse. But he was pale, and complained of almost constant headache. On the advice of Dr. Wallace, his eyes were during this time examined by Dr. Geo. W. Allyn, who found a low degree of astigmatism, and prescribed glasses.

On the morning of January 17th, last, the boy had a fainting spell before breakfast and became pale, weak and languid, complaining severely of the usual headache. At that date Dr. Wallace referred the parents to me, and the boy was brought to my office on the 18th of last January. A careful examination was made. Mentally the boy seemed clear, and gave prompt answers to all questions. His face was rather pale, the pupils widely dilated, contracting slightly to bright light. The tongue was extruded straight, and not much coated. The pulse about 80°, temperature normal. No abnormal motor or sensory phenomena were noted. The optic discs were normal. The urine contained neither albumin nor sugar.

The headache, of which he only complained, was referred to the vertex and forehead. The nature of the case was not entirely clear to me. The cause was obviously connected with the injury to the head, as he had been perfectly well up to that time. I therefore inclined to the opinion that the case was one of simple neuralgia, or else a slight injury to the cerebral membranes, perhaps a localized patch of congestion in the frontal region.

On this theory I prescribed small doses of sodium iodide and sodium bromide; also one grain of ergotin three times daily, and blistered each mastoid process. In three or four days the boy was brought back to my office without any improvement in the headache. The same treatment was continued excepting that ⅛ grain of protiodide of mercury was substituted for the ergotin. A mild galvanic current was passed for a few minutes from forehead to occiput. The parents were directed to discontinue this treatment in two days if he were not better, and to give tablets containing three grains of antipyrin every three hours. The day following this I was summoned in great haste by the father to meet Dr. Wallace, the former stating that he thought his son was dying. It seems that the lad complained of being sleepy and tired, and sitting on a rocking chair apparently went to sleep, but at once exhibited some general convulsive movements, and became wildly incoherent. When Dr. Wallace and I arrived, the boy was in bed and appeared rather bright and talked rationally. His pulse was about 90°, temperature normal. He complained, as usual, of the headache. When questioned as to the spell he had just had, he seemed to remember nothing about it. Very soon he was seized with one of these paroxysms, consisting of symmetrical clonic spasms of the forearms and hands, the same spasmodic movements passing to the lower extremities, the whole attack lasting scarcely a minute. There was apparently no loss of consciousness; no frothing of mouth; no change in the color or expression of face. At another time there would be a rapid turning of the head from side to side, or contortions of the entire body, or opisthotonus, accompanied by profane utterances, or other incoherent outbursts, terrifying and shocking his parents. We withdrew the iodide, increased the bromide to ten grains every three hours, and gave a little tr. of opium and aconite. Broths and other concentrated nourishment were given and accepted at frequent intervals. At our consultation the following day the boy was rather worse. The paroxysms, above described, had been more severe and frequent. The breathing at times had been irregular and jerky, diplopia had been observed. The head was occasionally retracted and bored into the pillow. The parents stated that he had been very "flighty"; that at times his eyes were glassy and upturned, and that they thought in one of these spells he would die. He had taken nourishment and slept fairly well, though during sleep there was much muscular twitching and catching respiration. When we entered the room the boy greeted us, and smiled and talked intelligently. We soon got him out of bed and tried to have him walk. His gait was very unsteady and he settled well back on his heels, inclining to fall backwards. But with assistance he walked some.

Replacing him in bed and retiring from the room we silently observed him through the slightly opened door. In a few moments he had one of the paroxysms above described. Gliding in swiftly, I pressed firmly on the supraorbital nerve. The boy immediately ceased his contortions, cried with pain, and said I had hurt him. We desired no further proof of the nature of the case, and the subsequent history confirmed the diagnosis. On appropriate treatment, of tonics, outdoor exercise, and the ignoring of his headache by the family, the boy gradually recovered, only once after having a hysterical attack, which was promptly arrested by the same means.

The fact that the patient was a boy, that he was so young, and that the headaches seemed to be of traumatic origin, quite misled us for a short time, as to the real nature of the trouble.

I will not detain the society with any remarks on hysterical affections in young children. They do, of course, occur in children of either sex, much younger than this one, but are, I believe, comparatively rare in our country. In France, nursery hysteria is by no means uncommon.

